# Clinical Significance of Serum Albumin and Implications of FcRn Inhibitor Treatment in IgG-Mediated Autoimmune Disorders

**DOI:** 10.3389/fimmu.2022.892534

**Published:** 2022-06-01

**Authors:** E. Sally Ward, Deborah Gelinas, Erwin Dreesen, Jolien Van Santbergen, Jan Terje Andersen, Nicholas J. Silvestri, Joseph E. Kiss, Darrell Sleep, Daniel J. Rader, John J. P. Kastelein, Els Louagie, Gestur Vidarsson, Isabel Spriet

**Affiliations:** ^1^Cancer Sciences Unit, Centre for Cancer Immunology, University of Southampton, Southampton, United Kingdom; ^2^Medical Affairs, argenx, Boston, MA, United States; ^3^Department of Pharmaceutical and Pharmacological Sciences, KU Leuven, Leuven, Belgium; ^4^Discovery, argenx, Ghent, Belgium; ^5^Department of Immunology, Oslo University Hospital Rikshospitalet, Oslo, Norway; ^6^Department of Pharmacology, University of Oslo, Oslo, Norway; ^7^Department of Neurology, University at Buffalo, Buffalo, NY, United States; ^8^Vitalant Northeast Division and Department of Medicine, University of Pittsburgh, Pittsburgh, PA, United States; ^9^Freelance Consultant, Nottingham, United Kingdom; ^10^Departments of Genetics and Medicine, Institute of Translational Medicine and Therapeutics, Perelman School of Medicine, University of Pennsylvania, Philadelphia, PA, United States; ^11^Department of Vascular Medicine, Genetics of Cardiovascular Disease, Academic Medical Center (AMC) of the University of Amsterdam, Amsterdam, Netherlands; ^12^Department of Experimental Immunohematology, Amsterdam University Medical Centers, University of Amsterdam, Amsterdam, Netherlands; ^13^Sanquin Research and Landsteiner Laboratory, Amsterdam University Medical Centers, University of Amsterdam, Amsterdam, Netherlands; ^14^Department of Clinical Pharmacology and Pharmacotherapy, KU Leuven, Leuven, Belgium; ^15^Pharmacy Department, University Hospitals Leuven, Leuven, Belgium

**Keywords:** albumin, autoimmune, FcRn, hypoalbuminemia, IgG, monoclonal antibody, serum protein

## Abstract

Serum albumin (SA), the most abundant soluble protein in the body, maintains plasma oncotic pressure and regulates the distribution of vascular fluid and has a range of other important functions. The goals of this review are to expand clinical knowledge regarding the functions of SA, elucidate effects of dysregulated SA concentration, and discuss the clinical relevance of hypoalbuminemia resulting from various diseases. We discuss potential repercussions of SA dysregulation on cholesterol levels, liver function, and other processes that rely on its homeostasis, as decreased SA concentration has been shown to be associated with increased risk for cardiovascular disease, hyperlipidemia, and mortality. We describe the anti-inflammatory and antioxidant properties of SA, as well as its ability to bind and transport a plethora of endogenous and exogenous molecules. SA is the primary serum protein involved in binding and transport of drugs and as such has the potential to affect, or be affected by, certain medications. Of current relevance are antibody-based inhibitors of the neonatal Fc receptor (FcRn), several of which are under clinical development to treat immunoglobulin G (IgG)-mediated autoimmune disorders; some have been shown to decrease SA concentration. FcRn acts as a homeostatic regulator of SA by rescuing it, as well as IgG, from intracellular degradation *via* a common cellular recycling mechanism. Greater clinical understanding of the multifunctional nature of SA and the potential clinical impact of decreased SA are needed; in particular, the potential for certain treatments to reduce SA concentration, which may affect efficacy and toxicity of medications and disease progression.

## Introduction

Serum albumin (SA) is the most abundant plasma protein in blood, and its well-known role in maintaining plasma oncotic pressure and regulating fluid distribution between vascular compartments (1–3) has historically overshadowed its other important functions. These include binding and transport of a diverse array of endogenous molecules and exogenous drugs, combined with its antioxidant and anti-inflammatory properties (1, 4). It is difficult to isolate a single function of SA, as its many roles are interconnected and collectively serve to maintain homeostasis in the body.

The objectives of this review are to expand clinical knowledge regarding the multiple functions of SA, elucidate the potential causes and effects of dysregulated SA concentration, and discuss the clinical relevance of hypoalbuminemia (defined as SA concentration <3.5 g/dL) (5) resulting from various diseases or certain drugs. The detailed mechanisms modulating drug interaction with SA in the presence and absence of endogenous and exogenous ligands have been described extensively elsewhere (6, 7) and will not be reviewed in detail here. Rather, we focus on several interconnected effects of SA on health and the potential clinical impact of SA concentration in patients with known risk factors, patients with chronic inflammatory disease or an autoimmune disorder, and patients with multiple comorbidities (e.g., dyslipidemia) (8). We also examine the potential for certain immunomodulatory neonatal Fc receptor (FcRn) inhibitors to reduce SA concentration and for reduced SA concentration to, in turn, affect the efficacy and toxicity of some medications. A better understanding of the interplay among SA concentration and endogenous and exogenous molecules will help clinicians recognize the potential for SA-related complications, elicit novel therapeutic insights, optimize treatment strategies and regimens, and improve quality of life for patients.

## Synthesis and Functions of Albumin

Albumin is exclusively synthesized by hepatocytes at a rate of roughly 150 mg/kg/day (9), meaning a person weighing 70 kg would synthesize approximately 10.5 g/day. The rate of synthesis is dependent on the body’s needs and on alterations in colloid osmotic pressure, as well as osmolality of the hepatic extravascular space (10). When a change in oncotic pressure in the hepatic vascular beds is detected by osmoreceptors in the hepatic interstitial matrix, the rate of albumin synthesis changes accordingly (10). It has been suggested that a healthy liver can boost synthesis of albumin by up to 3-fold in response to increased turnover or catabolism; however, there is scant evidence to support this widely held assumption (11).

Following synthesis in the liver, albumin is released in the extracellular space *via* exocytosis; ~40% is intravascular, and ~60% is distributed throughout organ/tissue interstitial spaces, predominantly in muscle, adipose and connective tissue, and skin (11). *Via* lymphatic vessels, there is movement of albumin between interstitial and intravascular spaces (1). Albumin has a high concentration in plasma, comprising roughly half of total protein content, and the high concentration together with a strong negative net charge are major contributors to the maintenance of plasma oncotic pressure (1). Interstitial concentration of albumin is lower than in serum and varies by anatomical location (1); concentrations of ~7 g/L in adipose tissue and ~13 g/L in skeletal muscle have been reported (12).

SA has a dominant role in binding and transport of numerous endogenous molecules, including thyroxine, bilirubin, amino acids, and fatty acids (FAs), throughout the body (1, 11). SA is considered the major plasma carrier of FAs, which play a critical role in generation and storage of energy for various cells and tissues throughout the body (13, 14). SA can accommodate a range of FA chain lengths among the 7 binding sites with differing affinities (FA1 through FA7) located across its 3 domains (15–17). SA binds cholesterol-containing vesicles and mediates transport of molecules between fibroblasts and other peripheral cells (18, 19). It also strongly binds and transports exogenous molecules—and their affinity for SA can influence drug behavior, potentially affecting the rate of delivery and the efficacy or toxicity of a drug (17, 20).

Its multiple binding sites provide an ideal platform for free radical scavenging, giving SA its robust anti-inflammatory and antioxidant properties (4, 21). SA also binds various inflammatory mediators and is involved in regulating the immune response in systemic inflammation (4). Because SA has been suggested to be responsible for >70% of total free radical–trapping activity, it constitutes the dominant antioxidant in the circulatory system (22). The reduced sulfhydryl group of Cys34 in domain I works as a free radical scavenger for multiple reactive oxygen species (ROS) and reactive nitrogen species (21, 23). SA can also bind free metal ions, thereby controlling their reactivity, limiting their availability, and decreasing ROS formation (21).

## FcRn Binding of Albumin and IgG

**Figure 1:**
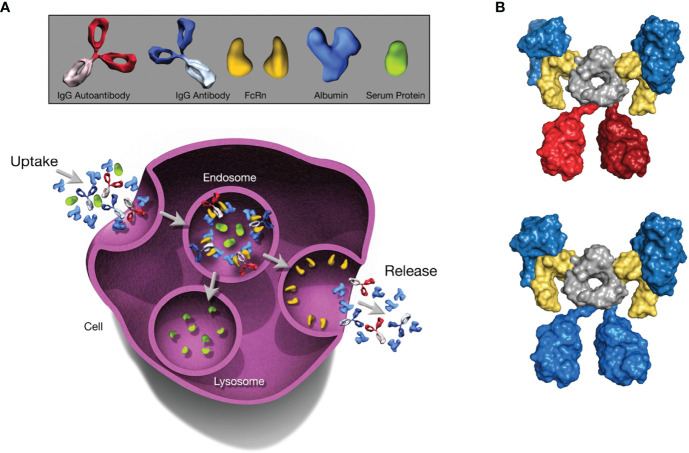
The Role of FcRn in Serum Albumin Regulation. The depicted processes of recycling and transcytosis collectively regulate SA concentration. **(A)** FcRn-mediated recycling and transcytosis rescue albumin and IgG from intracellular degradation. FcRn-albumin binding is critical for maintaining albumin homeostasis *via* scavenging, recycling, and transport of the FcRn-albumin-IgG complex through the endosomal recycling pathway. Subsequently, albumin and IgG antibodies are released into the extracellular space *via* exocytosis, whereas other proteins are degraded in lysosomes. **(B)** Molecular architecture of the complex between the extracellular region of FcRn (yellow), albumin (blue), and an IgG1 antibody (blue or red). The Fc moiety of an antibody (gray) can recruit 2 FcRn molecules. Simultaneously, each FcRn molecule can bind 1 additional albumin molecule (blue). All molecules are shown in surface representation. Figure generated with PyMOL using PDB entry 4N0U (30). Beta-2 microglobulin not shown. FcRn, neonatal Fc receptor; IgG, immunoglobulin G; SA, serum albumin.

In healthy humans, the half-life of SA is roughly 21 days; ultimately, ~84% is catabolized, with the remainder excreted *via* feces (~10%) and urine (~6%) (11). It should be noted that the amount of SA lost through urinary output is minimal, normally <20 mg/day (24). The unusually long half-life of SA is regulated by FcRn, a broadly expressed cellular receptor predominantly found in acidified endosomes (25, 26). This regulation occurs *via* pH-dependent engagement by the receptor (27), with binding at acidic endosomal pH (6.5-6.0) that becomes negligible at near-neutral pH (7.0-7.4), directing recycling or transcytosis across polarized cell layers and resulting in rescue of albumin from intracellular degradation (28, 29) (**Figure 1A**). Specifically, following fluid-phase pinocytosis, albumin binds FcRn at acidic pH within endosomes, then the FcRn-albumin complex recycles to the cell surface, where exposure to the neutral pH of the extracellular milieu triggers release of albumin into the circulation (31–33). Similarly to the endosomal pathway of immunoglobulin G (IgG) recycling (34), the albumin recycling process occurs in both hematopoietic and nonhematopoietic cells, particularly macrophages and endothelial cells, ensuring wide distribution of albumin throughout the body (35, 36). As such, in addition to liver synthesis, pH-dependent FcRn binding is critical for maintaining a high circulatory concentration of SA.

Because IgG antibodies also bind FcRn in a comparable pH-dependent manner, IgG has a similarly long half-life as albumin (25, 37, 38); however, IgG and albumin binding sites on FcRn are distinct and do not overlap (29, 38). In analyses of the structure of FcRn-albumin-antibody complexes, it has been shown that although binding of IgG and albumin to FcRn can occur simultaneously (30, 32) (**Figure 1B**), the fragment antigen-binding (Fab) arms of bound IgG may lead to steric clashes with the cell membrane, resulting in an orientation of FcRn with respect to the membrane that is less favorable for albumin binding (37). It has been proposed that, to overcome these clashes, IgG binds FcRn in a T-shaped conformation, which would be enabled by the highly flexible nature of the Fab arms (39). Studies have shown that the Fab arms of an IgG molecule can affect behavior in FcRn-mediated functions (40, 41), indicating that these effects vary for antibodies with different variable domain sequences and Fab arm flexibility (41, 42). The importance of FcRn for maintaining SA concentration has been demonstrated in multiple *in vitro* and preclinical studies in which FcRn deficiency decreased both half-life and concentration of SA (28, 35, 43, 44). FcRn knockout mice reportedly have an albumin catabolic rate twice that of normal mice, but synthesis rates only 20% higher than in normal mice and SA concentration that is 40% lower (45). FcRn directs newly synthesized albumin into the vascular space rather than into bile, a process that protects the liver from being damaged by albumin-bound toxins or drugs. Hepatic FcRn deficiency in mice has been shown to increase loss of SA into bile and influence the development of hypoalbuminemia (35). In addition, preclinical studies investigating maintenance of adequate SA concentration showed that FcRn expressed in the kidneys is also involved in preventing loss of SA. Albumin is filtered across the glomerulus and reclaimed by proximal tubule cells (46). FcRn in the apical area of the proximal tubule facilitates reclamation and mediates pH-dependent transcytosis with the albumin-binding cubilin-megalin complex (46, 47). FcRn-deficient mice were shown to excrete more SA into urine than did wild-type mice, and wild-type mice transplanted with a kidney lacking FcRn developed albuminuria (48).

## Drug Binding by Serum Albumin

SA is the principal plasma protein for the binding of drugs (49). The 3-dimensional structure of albumin comprises a single nonglycosylated polypeptide chain with 3 domains (I, II, and III), each consisting of 2 subdomains (A and B) and multiple reversible and irreversible binding sites (3, 20, 50). There are 2 main drug-binding sites, one in the IIA subdomain (Sudlow site I) and the other in the IIIA subdomain (Sudlow site II) (51). SA can also be harnessed to extend the half-life of drugs and reduce drug toxicity (23). Used as a carrier, SA joined to a therapeutic compound *via* covalent binding or noncovalent conjugation or fusion enables extension of the drug half-life and improved pharmacokinetic properties (52, 53).

Alterations to the structure of albumin or changes in its concentration in serum can alter drug-binding capacity, affecting pharmacokinetics and impacting therapeutic efficacy and/or side effects (7). Because only the unbound drug can interact with its target(s), alterations in SA concentration can lead to a drug being metabolized less or more quickly, influencing therapeutic effect (20). In addition, because low SA concentration leads to a reduction in available binding sites, a larger ratio of an administered SA-binding drug can be present as free drug, increasing the potential for side effects (54). The free fraction of a drug can be affected by concomitant administration of other drugs that also bind to SA, resulting in drug-drug interactions at the level of protein binding (17).

The long circulating half-life of SA increases its susceptibility to posttranslational modifications (PTMs), including glycation and oxidation (23, 52, 55). PTMs can alter the structure and/or function of SA (4, 56) and negatively impact its ability to bind FcRn, thereby decreasing its half-life (52, 57). Increased rates of glycation of SA, commonly seen in diabetes, can reduce drug-binding capacity, as can glycation-induced alterations to its molecular structure, independent of rate (3, 56). Increased glycation of SA has also been reported in patients with heart failure (HF), with highest levels in those with the most severe HF (58). Oxidation can similarly impact the binding properties of SA, and because oxidized SA is removed more quickly from circulation, overall availability and capacity for binding and transport are reduced (50). Increased S-thiolation (oxidation) of albumin has also been shown in the plasma of patients with HF, and level of thiolated SA may represent a viable marker for HF prognosis and diagnosis (59).

Many of the drugs in the standard treatment armamentarium for autoimmune disorders, including glucocorticoids such as prednisolone and methylprednisolone, as well as nonsteroidal immunosuppressants such as mycophenolic acid and tacrolimus, are highly protein bound, as are many common cardiovascular (CV) medications (**Table 1**). Patients with autoimmune conditions are frequently treated for CV comorbidities; in a sample of patients with myasthenia gravis (MG), dyslipidemia was the most common comorbidity (60%; 140 of 234 patients) (63). In addition, certain lipid-lowering drugs can affect SA concentration by increasing its urinary excretion. Statin treatment has been shown to be independently associated with microalbuminuria (64), defined as urinary excretion of 30 mg to 300 mg of albumin in a 24-hour period (65). It has been reported that approximately one-third of patients with MG are receiving statins (66) for the treatment of dyslipidemia, a prominent risk factor for CV disease (67). Accordingly, the potential for consequences from altered SA concentration in specific disease states may have greater clinical significance.

**Table 1:** Protein binding of medications used in autoimmune and cardiovascular diseases.

Disease Context	Drug Class	Drug	Protein Bound, %
**Autoimmune**			
	AChE inhibitors	Pyridostigmine*	~80
	Corticosteroids	Prednisone	<50
		Prednisolone	65-91
		Methylprednisolone	~76
		Dexamethasone	77
	Immunosuppressants	Azathioprine	30
		Mycophenolic acid^†^ Mycophenolate mofetil	98 97
		Cyclophosphamide^†^	20
		Methotrexate^†^	46.5-54
		Tacrolimus^†^	99
**Cardiovascular**			
	Statins	Simvastatin	~95
		Rosuvastatin	88
		Pravastatin	43-48
		Atorvastatin	>98
	Fibrates	Fenofibrate	99
		Ezetimibe	>90
	Beta blockers	Bisoprolol	~30
		Metoprolol	11
		Nebivolol	98
		Propranolol	~90
	CCBs	Nifedipine	92-98
	ACE inhibitors	Captopril	25-30
		Perindopril	10-20
		Enalapril	<50
	ATII inhibitors	Losartan	98
	Anticoagulants	Warfarin^†^ Apixaban	99 92-94
		Rivaroxaban	92-95
		Edoxaban	~55

ACE, angiotensin-converting enzyme; AChE, acetylcholinesterase; ATII, angiotensin II; CCBs, calcium channel blockers.

*Percentage for pyridostigmine is for albumin-specific binding (60); all others are general protein-binding percentages sourced from DrugBank (61).

^†^Indicates a drug that has been defined as having a narrow therapeutic index (62) by the US Food and Drug Administration.

The clinical impact of hypoalbuminemia is drug-specific and most well characterized for narrow therapeutic index drugs, defined by the US Food and Drug Administration (FDA) as drugs that can result in “serious therapeutic failures and/or adverse drug reactions that are life-threatening or result in persistent or significant disability or incapacity” due to minor differences in dose or blood concentration (62). For example, with valproic acid and phenytoin, it has been shown that reduced SA concentration can lead to a higher free fraction of these drugs (68), increasing the probability of (neuro)toxicity. The anticoagulants warfarin and rivaroxaban are both highly protein bound (99% and 92%-95%, respectively) (69) and hypoalbuminemia is associated with higher risk for overanticoagulation in patients taking warfarin (70). Lower SA level has also been associated with higher risk of bleeding events in rivaroxaban-treated patients; each decrease of 1.0 g/dL of SA in one inpatient population was shown to increase bleeding risk 4.4-fold (69). For many other drugs, both decreased efficacy and/or safety are still rather theoretical risks, based on mechanistic insights. To what extent these risks will be clinically relevant is a matter of ongoing debate.

## Pathophysiology of Serum Albumin Abnormalities

At present, SA concentration is normally assessed and monitored only in acute disease flares and following trauma, e.g., in hospitalized patients, although decreased SA concentration has been linked to worsening disease severity and increased mortality in multiple chronic conditions, including CV, metabolic, and autoimmune diseases and disorders (71, 72). Hypoalbuminemia may also result from primary chronic inflammatory disease, systemic inflammation, and kidney and liver disease, as well as from comorbid conditions (8).

The clinical implications of alterations in SA concentration seen with various disease states are not widely understood by clinicians (8) nor are the potential interactions with pharmacologic treatments, including the potential to enhance or hinder efficacy and increase toxicity of commonly prescribed drugs (7) or trigger or worsen comorbidities such as hyperlipidemia.

### Cardiovascular Disease and Dyslipidemia

**Figure 2:**
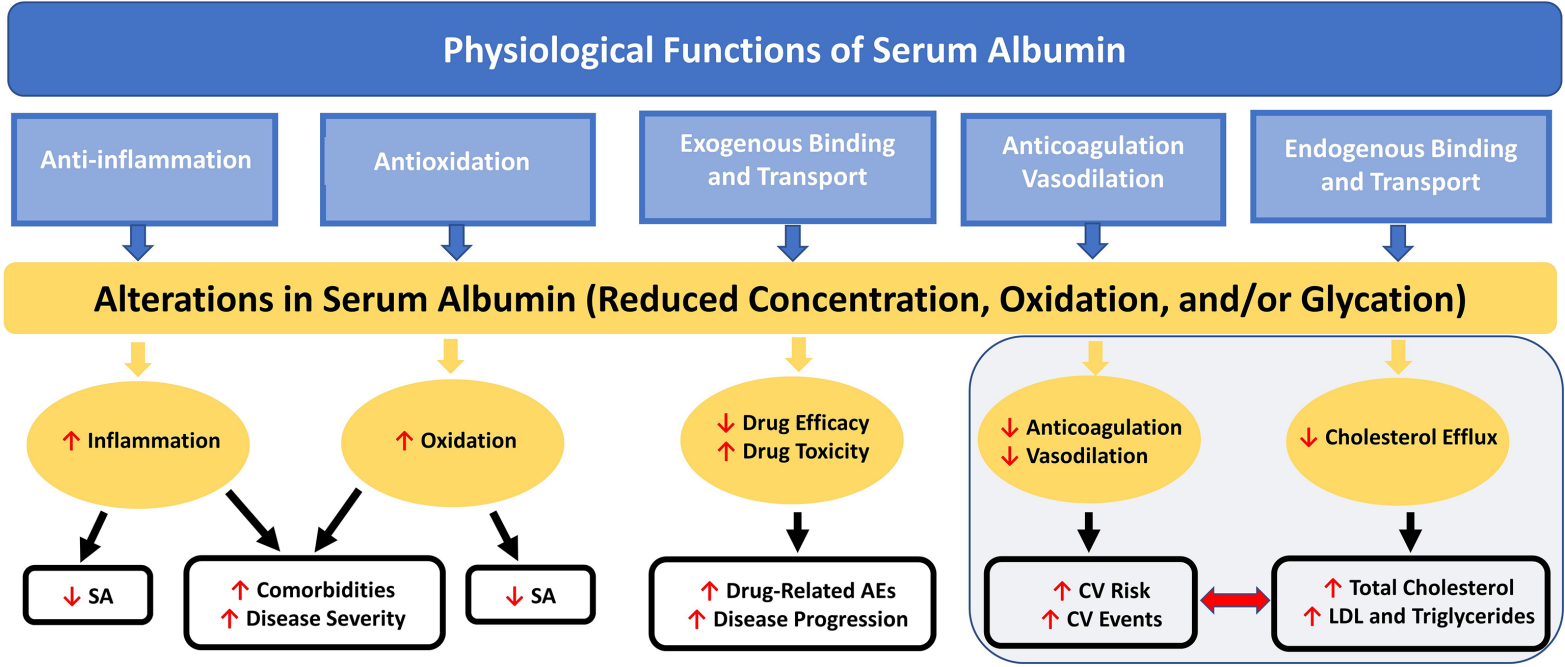
Nononcotic Pressure Functions of Serum Albumin and Consequences of Alterations in Concentration on Aspects of Health and Disease. Schematic representation of the interactive effects among the physiological functions (not including colloid oncotic pressure) of SA and the processes by which alterations in SA can lead to further decreases in SA concentration and to increases in disease severity and comorbidities; total cholesterol, LDL, and triglyceride levels; CV risk and events; and drug-related AEs. AE, adverse event; CV, cardiovascular; LDL, low-density lipoprotein; SA, serum albumin.

Hypoalbuminemia has emerged as a novel and potentially powerful prognostic marker in coronary artery disease (CAD) and appears to have predictive value for incidence of CV disease (73). Low SA concentration is associated with increased total and low-density lipoprotein (LDL) cholesterol levels, as well as increased CV mortality risk (73, 74). Low SA has also been related to impairments in fibrinolysis, vasodilatory ability, and anticoagulation and increased blood viscosity and vascular permeability (**Figure 2**), all factors associated with increased CV risk (18).

Low SA concentration, a characteristic of nephrotic syndrome, has been significantly negatively correlated with serum cholesterol levels and elevated total and LDL cholesterol in patients with this disorder, and with hypertriglyceridemia (74). Although the mechanisms are not yet well understood, it is known that in proteinuria, SA with less free fatty acid (FFA) content is lost; the remaining SA thus has higher FFA content and this imbalance contributes to development of hypertriglyceridemia (74). Microalbuminuria has also been shown to be an important CV risk factor in patients with diabetes or hypertension and in the general population (75, 76). Increased excretion of SA *via* urine, even at levels not meeting the standard for microalbuminuria, has been associated with increased incidence of all-cause and CV mortality (77).

The relationship between decreased SA concentration and elevated cholesterol has been explored in studies using albumin-deficient mice. Engineered *Alb*-/- mice were shown to have a generalized hyperlipidemic state in comparison to control mice (78). Total cholesterol (standard deviation [SD]) in the 2 strains of *Alb*-/- mice (B6 and Tg32) was 164.8 mg/dL (7.5) and 147.25 mg/dL (44.91), respectively, compared with their corresponding parental *Alb*+/+ mouse cohorts, 121.5 mg/dL (12.5) and 126.75 mg/dL (2.75), respectively (78). The LDL levels were similarly higher in the B6 and Tg32 line *Alb*-/- mice, 8.325 mg/dL (1.53) and 20.075 mg/dL (8.79), respectively, compared with the corresponding parental *Alb*+/+ mice, 4.025 mg/dL (0.70) and 5.35 mg/dL (0.94), respectively (78).

Reduction in SA concentration and plasma oncotic pressure can be compensated for by increased hepatic synthesis of albumin and apolipoprotein B-100 (apoB-100), among other serum proteins (79). Because SA is the primary transporter of FAs, dramatic reduction in SA can lead to increased FA transport by apoB-100, frequently resulting in dyslipidemia (80). Although the mechanisms of this are not well understood, total cholesterol levels are generally increased, mainly attributable to increased LDL levels, while high-density lipoprotein (HDL) levels remain the same or decrease (14). In addition, SA reduction can lead to defective cholesterol enrichment of HDL, because SA helps transfer free cholesterol from peripheral tissues to HDL particles. Similarly, in most reported cases of analbuminemia (congenital hypoalbuminemia), as well as in *Alb*-/- mouse models (78), hypercholesterolemia was primarily attributable to increased LDL concentration, with HDL concentration being less affected (80). Hypercholesterolemia resulting from lowered SA would, therefore, be expected to increase the risk for atherosclerotic complications. In addition, SA may play a role in facilitating cholesterol efflux from cells (81), a potentially cardioprotective mechanism.

### Acute and Chronic Diseases

SA is a negative acute-phase protein, meaning its concentration decreases in response to acute inflammatory response (18). In a prospective chart study of 30,732 hospitalized patients in 10 Israeli medical wards (January 2011-December 2013), hypoalbuminemia at admission was significantly associated with comorbid malignancy, hypertension, ischemic heart disease, and chronic kidney disease (71). When compared with the mean length of hospitalization for patients with normal SA concentration at admission (5±7 days), patients with mild (<2.5 g/dL) or marked (2.5-3.5 g/dL) hypoalbuminemia had longer stays (7±8 days and 9±11 days, respectively) in this study (71).

In severe liver disease, such as advanced cirrhosis, hypoalbuminemia can result from both decreased synthesis and increased posttranscriptional changes that alter structure and impair SA function (9). Such damage can adversely affect antioxidant, scavenging, immune-modulating, and endothelial protective functions and reduce the amount of SA that still has effective binding capacity (82).

Chronic disease or adverse lifestyle factors (eg, smoking, obesity) resulting in hypoalbuminemia can be compounded by aging itself, a slow yet inevitable inflammatory process (8). Hypoalbuminemia has been associated with increased risk of all-cause mortality (71) and microalbuminuria has been shown to be a strong independent predictor of mortality in a prospective general population cohort study (n = 40,856 questionnaire respondents; Netherlands) (83).

### Autoimmune Disorders

SA concentration is being investigated as a biomarker for severity, disease characteristics, and response to treatment in certain autoimmune disorders, including pemphigus vulgaris (PV) (84), Guillain-Barré syndrome (GBS) (85), and MG (86). Reduced SA concentration, commonly reported in patients with an autoimmune disorder, may additively worsen patient health and functioning, in part related to the amount of SA available to function as a critical antioxidant.

In one PV study, recently diagnosed patients (n=116) had significantly lower SA concentration compared to age- and sex-matched healthy controls (n=120; *P*<0.001) (84). In a Chinese report of patients with MG (n = 166) vs healthy controls (n=214), SA and serum creatinine concentrations were significantly lower in the patients with MG (*P*<0.001 for both) (86). In another report on these 166 inpatients with MG at a single hospital center in China (between 2009 and 2015), those who had a lower SA concentration had more-severe disease and a statistically significantly higher incidence of myasthenic crisis than those who had a higher SA concentration (*P*<0.05) (72).

Treatment with intravenous immunoglobulin (IVIg) may further decrease SA concentration. In a Dutch study of patients with GBS (n=174), after IVIg treatment, the percentage of patients with hypoalbuminemia increased from 12.8% to 34.5% of the study population (85). Low SA concentration, both pre- and posttreatment, was statistically significantly associated with poor clinical recovery (not able to walk 10 m independently and GBS disability score ≥2; *P*<0.001), independent of other clinical prognostic factors (85). History of IVIg treatment and hypoalbuminemia were statistically significantly (*P*<0.001) associated with poor outcomes (not achieving Myasthenia Gravis Foundation of America minimal manifestation) in a study of 104 patients with MG receiving treatment at a medical college in Japan between 2000 and 2017 (87).

## FcRn Inhibitor Treatments

Antibody-based drugs targeting the IgG binding site on FcRn are increasingly being explored to treat IgG-mediated autoimmune disorders in humans (88, 89); these drugs block the interaction of FcRn with endogenous IgG. Of the FcRn inhibitors in development, some have demonstrated, in preclinical, phase 1 (**Table 2**), and phase 2 trials (**Table 3**), an effect on SA concentration as well (89). It is currently unclear whether differences in effect on SA concentration may relate to differences in FcRn inhibitor design, such as format (full-length IgG vs Fc fragment), subclass, Fc effector functions, mode of binding, affinity, pH dependency, or binding epitope.

**Table 2:** Effect of FcRn inhibitors on serum albumin concentration in preclinical and phase 1 studies.

FcRn Inhibitor	Phase and Dosing Schedule	Effect on SA	Citation
**Rozanolixizumab** (Humanized IgG4 mAb)	• Preclinical RD in cynomolgus monkeys; 150 mg/kg IV q3d×13w or 50 or 150 mg/kg SC q3d in weeks 1, 6, and 10	Variable individual decreases (≤13% from baseline)	Smith B et al; *MAbs*; 2018 (90)
	• Phase 1 SAD (N=49); 1, 4, or 7 mg/kg or placebo; IV or SC	Variable individual decreases; not statistically significantly different from placebo	Kiessling P et al; *Sci Transl Med*; 2017 (NCT02220153) (91)
**Nipocalimab** (Fully human aglycosylated IgG1 mAb)	• Preclinical in cynomolgus monkeys	No effect	Ling LE et al; *Am Soc Hematol*; 2015 (92)
	• Phase 1 SAD cohort (n=34); 0.3, 3, 10, 20, or 60 mg/kg or placebo; IV	Mild, asymptomatic reductions	Ling LE et al; *Clin Pharmacol Ther*; 2019 (93)
	• Phase 1 MAD cohort (n=16); 15 or 30 mg/kg or placebo; IV qw×4w	Up to 22% reduction from baseline	Ling LE et al; *Clin Pharmacol Ther*; 2019 (93)
**Orilanolimab** (Humanized IgG4 mAb)	• Preclinical RD in cynomolgus monkeys; 10, 30, or 100 mg/kg; IV qw×5	No effect	Blumberg LJ et al; *Sci Adv*; 2019 (94)
	• Phase 1 SAD (n=31); 1, 3, 10, or 30 mg/kg or placebo; IV	No effect	Blumberg LJ et al; *Sci Adv*; 2019 (NCT03643627) (94)
**Batoclimab** (Fully human aglycosylated IgG1 mAb)	• Phase 1 SAD cohort (n=24); 340, 510, or680 mg or placebo; SC injection	Reversible reductions ≤10% from baseline	Yap DYH et al; *Clin Transl Sci*; 2021 (NCT03971916) (95)
	• Phase 1 MAD cohort (n=20); 340 mg or 680 mg or placebo; SC injection qw×4w	Reversible, dose-dependent decreases: 20% below baseline after 340 mg qw×4w and 31% below baseline after 680 mg qw×4w	Collins J et al; *Neurology*; 2019 (96)
**Efgartigimod** (Modified Fc fragment)	• Preclinical in cynomolgus monkeys	Not reported	Ulrichts P et al; *J Clin Investig*; 2018 (97)
	• Phase 1 SAD cohort (n=30); 0.2, 2, 10, 25, or 50 mg/kg or placebo; IV	No decrease	Ulrichts P et al; *J Clin Investig*; 2018 (NCT03457649) (97)
	• Phase 1 MAD cohort (n=32); 10 mg/kg q4d×6, 10 mg/kg q7d×4, 25 mg/kg q7d×4, or placebo; IV	No decrease	Ulrichts P et al; *J Clin Investig*; 2018 (NCT03457649) (97)

IgG, immunoglobulin G; IV, intravenous; mAb, monoclonal antibody; MAD, multiple ascending dose; RD, repeated dose; SAD, single ascending dose; SC, subcutaneous; qw, every week; q3d, every 3 days; q3w, every 3 weeks; q7d, every 7 days; w, week.

**Table 3:** Effect of FcRn inhibitors on serum albumin concentration and cholesterol in phase 2 and phase 3 studies.

FcRn Inhibitor	Phase	Treatment and Dosing Schedule	Effect on SA	Impact on Cholesterol	Citation
**Rozanolixizumab** (Humanized IgG4 mAb)	Phase 2 in ITP (N=66)	SAD of 15 mg/kg or 20 mg/kg, MAD of 4 mg/kg qw×5w, 7 mg/kg qw×3w, or 10 mg/kg qw×2w; SC infusion	Max mean decrease 4.5% from baseline (WNL)	Not reported	Robak T et al; *Blood Adv*; 2020 (NCT02718716) (98)
	Phase 2a in MG (N=43)	Period 1: 7 mg/kg qw×3w or placebo; SC infusion (2-week washout)	Not reported	Not reported	Bril V et al; *Neurology*; 2021 (NCT03052751) (99)
		Period 2: 4 mg/kg, 7 mg/kg, or placebo qw×3w; SC infusion			
**Nipocalimab** (Fully human aglycosylated IgG1 mAb)	Phase 2 in gMG (N=68)	SAD of 5 mg/kg qw×4, 30 mg/kg qw×4, 60 mg/kg q2w×5, SD of 60 mg/kg, or placebo; IV	Reductions reported; greatest reductions in 60-mg/kg q2w group	Not reported	Wolfe GI et al; *J Neurol Sci*; 2021 (NCT03772587) (89)
**Orilanolimab** (Humanized IgG4 mAb)	Phase 1b/2 in PV (N=8)	10 mg/kg qw×5w; IV	No noteworthy effects reported	Not reported	Werth VP et al; *J Investig Derm*; 2021 (NCT03075904) (100)
**Batoclimab** (Fully human aglycosylated IgG1 mAb)	Phase 2 in gMG (N=17)	340 mg, 680 mg, or placebo qw×6; SC injection	Reductions of 16% from baseline in 340-mg group and 26% in 680-mg group	Not reported	Wolfe GI et al; *J Neurol Sci*; 2021 (NCT03863080) (89)
	Phase 2b in TED (N=65)	255 mg, 340 mg, 680 mg, or placebo qw×12w; SC injection	Not reported (trial voluntarily paused)	Elevated total cholesterol and LDL	Wolfe GI et al; *J Neurol Sci*; 2021 (89) Men CJ et al; *Ther Adv Ophthalmol*; 2021 (NCT03938545) (101)
**Efgartigimod** (Modified Fc fragment)	Phase 2 in ITP (N=38)	5 mg/kg, 10 mg/kg, or placebo qw×4w; IV	Similar to placebo	Not reported	Newland AC et al; *Am J Hematol*; 2020 (NCT03102593) (102)
	Phase 2 in PV/PF (N=34)	10 mg/kg or 25 mg/kg qw×4w; IV	Transient increases (WNL)	No impact (n=11 patients in cohort 4)	Goebler M et al; *Br J Dermatol*; 2021 (NCT03334058) (103)
	Phase 3 in gMG (N=167)	10 mg/kg or placebo qw×4w; IV	No decrease	Not reported	Howard JF Jr; *Lancet Neurol*; 2021 (NCT03334058) (104)

IgG, immunoglobulin G; ITP, immune thrombocytopenia; IV, intravenous; gMG, generalized myasthenia gravis; mAb, monoclonal antibody; MAD, multiple ascending dose; MG, myasthenia gravis; PF, pemphigus foliaceus; PV, pemphigus vulgaris; SAD, single ascending dose; SC, subcutaneous; SD, single dose; TED, thyroid eye disease; qw, every week; q2w, every 2 weeks; w, week; WNL, within normal limits (3.5-5.0 g/dL).

Rozanolixizumab/UCB7655 (UCB Biopharma), a humanized IgG4 monoclonal antibody (mAb), showed modest decreases in mean SA concentration in a phase 1 study in healthy adult volunteers treated with a low-dose regimen (91). In a phase 2 study of rozanolixizumab in adult patients with primary immune thrombocytopenia (ITP), a maximum mean decrease in SA concentration of 4.5% was observed with the 20-mg/kg dose at day 8, which returned to baseline levels (98).

A phase 1 study of nipocalimab/M281 (Momenta/Janssen), a fully human IgG1 mAb (aglycosylated to reduce effector functions), showed asymptomatic transient reductions in SA concentration (up to 22%) in patients in the single ascending and multiple dose phases of the study (89, 93). In a phase 2 study in generalized MG (gMG), reductions in SA were observed across 4 intravenous (IV) dosing regimens (5 mg/kg every 4 weeks, 30 mg/kg every 4 weeks, 60 mg/kg every 2 weeks, and 60 mg/kg single dose) over 8 weeks of treatment; the largest reduction was observed in the group receiving 60 mg/kg every 2 weeks (83).

Orilanolimab/ALX1380 [AstraZeneca/Alexion (formerly SYNT001, Syntimmune)], a humanized IgG4 mAb, was reported to have no effect on SA concentration in a phase 1 healthy volunteer study using single ascending doses (94). In a phase 1b/2 study of orilanolimab in patients with PV, no noteworthy changes in SA concentration after 5 weekly IV doses of 10 mg/kg were reported (100).

Administration of batoclimab/IMVT-1401/HBM9161 (Immunovant), a fully human IgG1 mAb with Fc mutations (L234A/L235A) to reduce effector functions, resulted in reversible, asymptomatic, dose-dependent reductions in SA concentration, to 20% below baseline by day 28 after 4 weekly subcutaneous (SC) doses of 340 mg and to 31% below baseline after 4 weekly SC doses of 680 mg, in a phase 1 healthy volunteer study (96). In another phase 1 study of batoclimab in healthy volunteers, a single SC dose of 680 mg resulted in transient reductions of SA up to 10% below baseline at day 11 (95). A phase 2 study in gMG showed reductions in SA concentration of 16% with 6 weekly SC doses of 340 mg and 26% with 6 weekly SC doses of 680 mg (89). A phase 2b clinical trial of batoclimab as treatment for thyroid eye disease was paused when a 12-week assessment showed elevated total and LDL cholesterol levels in active-treatment patients (89, 101).

Efgartigimod alfa (argenx), recently approved by the FDA for treatment of gMG (105), is an engineered Fc fragment derived from human IgG1 that blocks FcRn recycling of IgG by binding naturally *via* its Fc domain to FcRn (97, 106). SA concentration was not decreased by efgartigimod in a phase 1 trial in healthy volunteers (97). In a phase 2 study of efgartigimod in patients with ITP randomly assigned to receive 4 weekly IV infusions of placebo or efgartigimod at a dose of 5 mg/kg or 10 mg/kg, changes in SA concentration from baseline to 3 days after final infusion were similar for the placebo and treated groups (102). In a phase 2 trial of up to 34 weeks of efgartigimod treatment in PV/pemphigus foliaceus, average SA concentration increased slightly and transiently, within normal limits, and returned to baseline levels after treatment was stopped (103). Total serum cholesterol and LDL levels, as measured in 1 cohort, remained within normal limits across all active study and follow-up assessment points (103). In the phase 3 ADAPT trial of efgartigimod in gMG, no decreases in SA concentration or increases in cholesterol were seen (104).

## Discussion

The clinical importance of SA concentration and the relationship of SA to disease, in combination with therapeutics, is complex. Increasing evidence suggests it may be useful to monitor SA concentration in patients with diseases or disorders that affect its steady-state concentration. Dysregulation of SA in CV disease, diabetes, and certain autoimmune disorders, including MG and GBS, can affect multiple body systems. Oxidation and glycation of SA, resulting from disease processes, as well as from aging, complicate the clinical picture. Awareness of these changes could help clinicians better manage disease.

Although the published literature is limited, hypoalbuminemia in autoimmune disorders has been associated with increased disease severity. Because SA is largely responsible for the antioxidant capacity of serum, the decreased SA concentration commonly seen in patients with autoimmune disorders may contribute to the underlying inflammatory processes. Additionally, many of the primary medications, including prednisolone, methylprednisolone, and the nonsteroidal immunosuppressant mycophenolate mofetil, used to treat various autoimmune diseases are protein bound. Therefore, their efficacy and safety could be affected by reductions in available SA binding sites, leading to increased drug clearance.

Some newer drugs on the horizon for the treatment of autoimmune disorders also have SA-related effects. FcRn inhibitors targeting the IgG binding site on the receptor are not intended to decrease SA, only to accelerate clearance of endogenous IgG. In clinical trials, however, some IgG mAbs that reduce IgG levels by inhibiting FcRn-IgG interactions have had the off-target effect of concomitantly decreasing SA concentration. Important questions remain regarding the link between FcRn inhibitors and SA. Flow cytometry and microscopy data have shown that higher affinity and/or avidity and/or lower pH-dependent binding to FcRn of an anti-FcRn antibody, relative to the engineered Fc fragment efgartigimod, results in greater retention in FcRn-positive compartments, as well as increased lysosomal accumulation (97). Upon investigation of intracellular trafficking of FcRn by immune complexes, it was shown that cross-linking of FcRn diverted the majority of the cross-linked receptors to lysosomes (107). These findings suggest that the different binding properties of FcRn-specific antibodies and an engineered Fc fragment (with increased affinity for FcRn) might modulate the behavior of FcRn within cells, leading to possible effects on SA recycling.

An increased understanding of the nononcotic functions of SA, especially during this period of accelerated drug development in MG (89, 108), may help improve the risk/benefit assessment and selection of optimal therapeutic regimen for a wide array of disease conditions and patient types. Although increased catabolism or excretion of SA is partly compensated for by increased synthesis, resulting in a milder net loss, stimulating liver synthesis of proteins such as apoB-100 and fibrinogen can result in comorbidities, including dyslipidemia. Greater clinical attention to SA concentration is warranted for older patients, patients on multiple drugs that could potentially affect or be affected by SA concentration, and patients with multiple comorbidities.

## Future Perspectives

As FcRn inhibitors become used more widely in clinical trials, the potential for mild to moderate decreases in SA concentration to have subsequent impacts on lipid panels and CV risk may become increasingly apparent as additional patient populations are treated. Because many of these patients will be older and may have comorbidities, the importance of a fuller understanding by healthcare providers of the role of SA in healthy and disease states will grow more urgent. The most obvious clinical indications are the various IgG-mediated auto- and alloimmune- diseases, meaning these patients may be inherently more susceptible to treatment side effects resulting from lowered SA concentration. Fortunately, several candidate FcRn inhibitors have shown little or no effect on SA concentration and might, therefore, be safer in a broader array of disease settings.

## Conclusions

Increased awareness is needed regarding the clinical importance of monitoring plasma SA concentration in patients with diseases or disorders known to correlate with decreased SA concentration and/or who are being treated with drugs that may lower SA concentration, independently or in combination with concomitantly administered drugs. As more data become available, the question whether clinicians should routinely evaluate SA concentration to aid in monitoring disease progression and decreasing the potential for side effects and adverse events could gain traction. As such, SA concentration may become an increasingly important consideration in the design and outcomes of clinical trials using FcRn inhibitors, as well as potential future treatment with FcRn inhibitors that are associated with decreases in SA concentration.

## Author Contributions

ESW – provided substantial contributions to the design of the work and interpretation of current data based on expertise in FcRn-SA-IgG interaction and to figure development; drafted sections of the work and revised the manuscript critically for important intellectual content; shares first authorship; DG – provided substantial contributions to the design of the work and interpretation of current data based on her clinical expertise in the neuromuscular field; drafted sections of the work and revised the manuscript critically for important intellectual content; shares first authorship; ED – provided content on exogenous drug binding and revised the manuscript critically for important intellectual content; JVS – provided original concept, initial research, and content; revised the manuscript critically for important intellectual content; JTA – provided expertise in and content on FcRn-SA-IgG interaction; revised the manuscript critically for important intellectual content; NS – provided clinical and application expertise in neuromuscular disorders and revised the manuscript critically for important intellectual content; JEK – provided clinical expertise on albumin in hematology; revised the manuscript critically for important intellectual content; DS – guided understanding on the interaction of human albumin with FcRn and other proteins; revised the manuscript critically for important intellectual content; DR – provided clinical and research expertise on albumin’s relation to cholesterol metabolism; provided text and content for new direction of the manuscript; revised the manuscript critically for important intellectual content; JJPK – provided expertise on cholesterol metabolism for new direction of the manuscript and reframed the discussion of albumin’s potential role in cholesterol metabolism; revised the manuscript critically for important intellectual content; EL – developed original concept for manuscript and provided nonclinical expertise on FcRn; provided content and critical revision of manuscript for important intellectual content; GV – provided expertise on FcRn-SA-IgG interaction; revised the manuscript critically for important intellectual content; IS – provided content on exogenous drug binding and revised the manuscript critically for important intellectual content. All authors contributed to the article and approved the submitted version.

## Conflict of Interest

DG, EL, and JVS are employees of argenx, the manufacturer of efgartigimod (FDA approved in gMG and under investigation in primary immune thrombocytopenia, pemphigus vulgaris and foliaceus, chronic inflammatory demyelinating polyneuropathy, bullous pemphigoid, and idiopathic inflammatory myopathy [myositis]). JTA has received funding from Syntimmune and argenx as part of fee-for-service agreements. ED is a postdoctoral research fellow of the Research Foundation – Flanders (grant number 12X9420N). JEK has participated on the Medical Advisory Committee of Sanofi Genzyme and Plasma Advisory Committee of Haemonetics. DR is the founder of Staten Biotechnology. NS is a member of the scientific advisory board and a speaker for argenx. IS, is supported by the Clinical Research Fund of the University Hospitals Leuven. ESW may receive royalties from patents owned by the UK Medical Research Council, UT Southwestern Medical Center, and Texas A&M University, and has financial interests in argenx, Astero BioPharma LLC, Astero Erado Inc., and Astero Technologies LLC. GV is a paid consultant for argenx.

The remaining authors declare that the research was conducted in the absence of any commercial or financial relationships that could be construed as a potential conflict of interest.

The development (medical writing and editorial support) of this manuscript received funding from argenx. Authors who are not affiliated with argenx were not paid for their contributions to this work. The funder was involved in conception of design, interpretation of data, writing of this article, and decision to submit it for publication.

## Publisher’s Note

All claims expressed in this article are solely those of the authors and do not necessarily represent those of their affiliated organizations, or those of the publisher, the editors and the reviewers. Any product that may be evaluated in this article, or claim that may be made by its manufacturer, is not guaranteed or endorsed by the publisher.

## References

[1] Evans TW. Review Article: Albumin as a Drug–Biological Effects of Albumin Unrelated to Oncotic Pressure. Aliment Pharmacol Ther (2002) 16(Suppl 5):6–11. doi: 10.1046/j.1365-2036.16.s5.2.x 12423448

[2] Merlot AM Kalinowski DS Richardson DR. Unraveling the Mysteries of Serum Albumin-More Than Just a Serum Protein. Front Physiol (2014) 5:299. doi: 10.3389/fphys.2014.00299 25161624 PMC4129365

[3] Rondeau P Bourdon E. The Glycation of Albumin: Structural and Functional Impacts. Biochimie (2011) 93(4):645–58. doi: 10.1016/j.biochi.2010.12.003 21167901

[4] Sun L Yin H Liu M Xu G Zhou X Ge P. Impaired Albumin Function: A Novel Potential Indicator for Liver Function Damage? Ann Med (2019) 51(7-8):333–44. doi: 10.1080/07853890.2019.1693056 PMC7877890 31714153

[5] Zhang Z Pereira SL Luo M Matheson EM. Evaluation of Blood Biomarkers Associated With Risk of Malnutrition in Older Ddults: A Systematic Review and Meta-Analysis. Nutrients (2017) 9(8):829. doi: 10.3390/nu9080829 PMC5579622 28771192

[6] Leboffe L di Masi A Polticelli F Trezza V Ascenzi P. Structural Basis of Drug Recognition by Human Serum Albumin. Curr Med Chem (2020) 27(30):4907–31. doi: 10.2174/0929867326666190320105316 30894098

[7] Yamasaki K Chuang VT Maruyama T Otagiri M. Albumin-Drug Interaction and its Clinical Implication. Biochim Biophys Acta (2013) 1830(12):5435–43. doi: 10.1016/j.bbagen.2013.05.005 23665585

[8] Soeters PB Wolfe RR Shenkin A. Hypoalbuminemia: Pathogenesis and Clinical Significance. JPEN J Parenter Enteral Nutr (2019) 43(2):181–93. doi: 10.1002/jpen.1451 PMC7379941 30288759

[9] Carvalho JR Verdelho Machado M. New Insights About Albumin and Liver Disease. Ann Hepatol (2018) 17(4):547–60. doi: 10.5604/01.3001.0012.0916 29893696

[10] Wingfield W. Fluid and Electrolyte Therapy. In: Raffe MR, editor. The Veterinary ICU Book. Jackson, WY: Teton New Media (2002). p. 166–88.

[11] Levitt DG Levitt MD. Human Serum Albumin Homeostasis: A New Look at the Roles of Synthesis, Catabolism, Renal and Gastrointestinal Excretion, and the Clinical Value of Serum Albumin Measurements. Int J Gen Med (2016) 9:229–55. doi: 10.2147/IJGM.S102819 PMC4956071 27486341

[12] Ellmerer M Schaupp L Brunner GA Sendlhofer G Wutte A Wach P. Measurement of Interstitial Albumin in Human Skeletal Muscle and Adipose Tissue by Open-Flow Microperfusion. Am J Physiol Endocrinol Metab (2000) 278(2):E352–6. doi: 10.1152/ajpendo.2000.278.2.E352 10662720

[13] van der Vusse GJ. Albumin as Fatty Acid Transporter. Drug Metab Pharmacokinet (2009) 24(4):300–7. doi: 10.2133/dmpk.24.300 19745557

[14] Vaziri ND. Disorders of Lipid Metabolism in Nephrotic Syndrome: Mechanisms and Consequences. Kidney Int (2016) 90(1):41–52. doi: 10.1016/j.kint.2016.02.026 27165836 PMC5812444

[15] Coverdale JPC Khazaipoul S Arya S Stewart AJ Blindauer CA. Crosstalk Between Zinc and Free Fatty Acids in Plasma. Biochim Biophys Acta Mol Cell Biol Lipids (2019) 1864(4):532–42. doi: 10.1016/j.bbalip.2018.09.007 PMC6372834 30266430

[16] Ha CE Bhagavan NV. Novel Insights Into the Pleiotropic Effects of Human Serum Albumin in Health and Disease. Biochim Biophys Acta (2013) 1830(12):5486–93. doi: 10.1016/j.bbagen.2013.04.012 23602811

[17] Fasano M Curry S Terreno E Galliano M Fanali G Narciso P. The Extraordinary Ligand Binding Properties of Human Serum Albumin. IUBMB Life (2005) 57(12):787–96. doi: 10.1080/15216540500404093 16393781

[18] Chien SC Chen CY Lin CF Yeh HI. Critical Appraisal of the Role of Serum Albumin in Cardiovascular Disease. Biomark Res (2017) 5:31. doi: 10.1186/s40364-017-0111-x 29152305 PMC5681838

[19] Peng L Minbo H Fang C Xi L Chaocan Z. The Interaction Between Cholesterol and Human Serum Albumin. Protein Pept Lett (2008) 15(4):360–4. doi: 10.2174/092986608784246542 18473948

[20] Yang F Zhang Y Liang H. Interactive Association of Drugs Binding to Human Serum Albumin. Int J Mol Sci (2014) 15(3):3580–95. doi: 10.3390/ijms15033580 PMC3975355 24583848

[21] Taverna M Marie AL Mira JP Guidet B. Specific Antioxidant Properties of Human Serum Albumin. Ann Intensive Care (2013) 3(1):4. doi: 10.1186/2110-5820-3-4 23414610 PMC3577569

[22] Roche M Rondeau P Singh NR Tarnus E Bourdon E. The Antioxidant Properties of Serum Albumin. FEBS Lett (2008) 582(13):1783–7. doi: 10.1016/j.febslet.2008.04.057 18474236

[23] Arroyo V Garcia-Martinez R Salvatella X. Human Serum Albumin, Systemic Inflammation, and Cirrhosis. J Hepatol (2014) 61(2):396–407. doi: 10.1016/j.jhep.2014.04.012 24751830

[24] Nicholson JP Wolmarans MR Park GR. The Role of Albumin in Critical Illness. Br J Anaesth (2000) 85(4):599–610. doi: 10.1093/bja/85.4.599 11064620

[25] Ward ES Ober RJ. Chapter 4 Multitasking by Exploitation of Intracellular Transport Functions. Adv Immunol (2009) 103:77–115. doi: 10.1016/s0065-2776(09)03004-1 19755184 PMC4485553

[26] Pyzik M Rath T Lencer WI Baker K Blumberg RS. FcRn: The Architect Behind the Immune and Nonimmune Functions of IgG and Albumin. J Immunol (2015) 194(10):4595–603. doi: 10.4049/jimmunol.1403014 PMC4451002 25934922

[27] Sand KM Dalhus B Christianson GJ Bern M Foss S Cameron J. Dissection of the Neonatal Fc Receptor (FcRn)-Albumin Interface Using Mutagenesis and Anti-FcRn Albumin-Blocking Antibodies. J Biol Chem (2014) 289(24):17228–39. doi: 10.1074/jbc.M113.522565 PMC4059163 24764301

[28] Chaudhury C Mehnaz S Robinson JM Hayton WL Pearl DK Roopenian DC. The Major Histocompatibility Complex-Related Fc Receptor for IgG (FcRn) Binds Albumin and Prolongs its Lifespan. J Exp Med (2003) 197(3):315–22. doi: 10.1084/jem.20021829 PMC2193842 12566415

[29] Andersen JT Dee Qian J Sandlie I. The Conserved Histidine 166 Residue of the Human Neonatal Fc Receptor Heavy Chain is Critical for the pH-Dependent Binding to Albumin. Eur J Immunol (2006) 36(11):3044–51. doi: 10.1002/eji.200636556 17048273

[30] Oganesyan V Damschroder MM Cook KE Li Q Gao C Wu H. Structural Insights Into Neonatal Fc Receptor-Based Recycling Mechanisms. J Biol Chem (2014) 289(11):7812–24. doi: 10.1074/jbc.M113.537563 PMC3953293 24469444

[31] Larsen MT Rawsthorne H Schelde KK Dagnaes-Hansen F Cameron J Howard KA. Cellular Recycling-Driven *In Vivo* Half-Life Extension Using Recombinant Albumin Fusions Tuned for Neonatal Fc Receptor (FcRn) Engagement. J Control Release (2018) 287:132–41. doi: 10.1016/j.jconrel.2018.07.023 30016735

[32] Schmidt MM Townson SA Andreucci AJ King BM Schirmer EB Murillo AJ. Crystal Structure of an HSA/FcRn Complex Reveals Recycling by Competitive Mimicry of HSA Ligands at a pH-Dependent Hydrophobic Interface. Structure (2013) 21(11):1966–78. doi: 10.1016/j.str.2013.08.022 24120761

[33] Sand KM Bern M Nilsen J Noordzij HT Sandlie I Andersen JT. Unraveling the Interaction Between FcRn and Albumin: Opportunities for Design of Albumin-Based Therapeutics. Front Immunol (2014) 5:682. doi: 10.3389/fimmu.2014.00682 25674083 PMC4306297

[34] Ober RJ Martinez C Vaccaro C Zhou J Ward ES. Visualizing the Site and Dynamics of IgG Salvage by the MHC Class I-Related Receptor, FcRn. J Immunol (2004) 172(4):2021–9. doi: 10.4049/jimmunol.172.4.2021 14764666

[35] Pyzik M Rath T Kuo TT Win S Baker K Hubbard JJ. Hepatic FcRn Regulates Albumin Homeostasis and Susceptibility to Liver Injury. Proc Natl Acad Sci USA (2017) 114(14):E2862–E71. doi: 10.1073/pnas.1618291114 PMC5389309 28330995

[36] Challa DK Wang X Montoyo HP Velmurugan R Ober RJ Ward ES. Neonatal Fc Receptor Expression in Macrophages is Indispensable for IgG Homeostasis. MAbs (2019) 11(5):848–60. doi: 10.1080/19420862.2019.1602459 PMC6601554 30964743

[37] Pyzik M Sand KMK Hubbard JJ Andersen JT Sandlie I Blumberg RS. The Neonatal Fc Receptor (FcRn): A Misnomer? Front Immunol (2019) 10:1540. doi: 10.3389/fimmu.2019.01540 31354709 PMC6636548

[38] Chaudhury C Brooks CL Carter DC Robinson JM Anderson CL. Albumin Binding to FcRn: Distinct From the FcRn-IgG Interaction. Biochemistry (2006) 45(15):4983–90. doi: 10.1021/bi052628y 16605266

[39] Booth BJ Ramakrishnan B Narayan K Wollacott AM Babcock GJ Shriver Z. Extending Human IgG Half-Life Using Structure-Guided Design. MAbs (2018) 10(7):1098–110. doi: 10.1080/19420862.2018.1490119 PMC6204840 29947573

[40] Schlothauer T Rueger P Stracke JO Hertenberger H Fingas F Kling L. Analytical FcRn Affinity Chromatography for Functional Characterization of Monoclonal Antibodies. MAbs (2013) 5(4):576–86. doi: 10.4161/mabs.24981 PMC3906311 23765230

[41] Schoch A Kettenberger H Mundigl O Winter G Engert J Heinrich J. Charge-Mediated Influence of the Antibody Variable Domain on FcRn-Dependent Pharmacokinetics. Proc Natl Acad Sci USA (2015) 112(19):5997–6002. doi: 10.1073/pnas.1408766112 25918417 PMC4434771

[42] Stapleton NM Brinkhaus M Armour KL Bentlage AEH de Taeye SW Temming AR. Reduced FcRn-Mediated Transcytosis of IgG2 Due to a Missing Glycine in its Lower Hinge. Sci Rep (2019) 9(1):7363. doi: 10.1038/s41598-019-40731-2 31089170 PMC6517591

[43] Montoyo HP Vaccaro C Hafner M Ober RJ Mueller W Ward ES. Conditional Deletion of the MHC Class I-Related Receptor FcRn Reveals the Sites of IgG Homeostasis in Mice. Proc Natl Acad Sci USA (2009) 106(8):2788–93. doi: 10.1073/pnas.0810796106 PMC2650344 19188594

[44] Bern M Nilsen J Ferrarese M Sand KMK Gjølberg TT Lode HE. An Engineered Human Albumin Enhances Half-Life and Transmucosal Delivery When Fused to Protein-Based Biologics. Sci Transl Med (2020) 12(565):eabb0580. doi: 10.1126/scitranslmed.abb0580 33055243

[45] Kim J Bronson CL Hayton WL Radmacher MD Roopenian DC Robinson JM. Albumin Turnover: FcRn-Mediated Recycling Saves as Much Albumin From Degradation as the Liver Produces. Am J Physiol Gastrointest Liver Physiol (2006) 290(2):G352–60. doi: 10.1152/ajpgi.00286.2005 16210471

[46] Dickson LE Wagner MC Sandoval RM Molitoris BA. The Proximal Tubule and Albuminuria: Really! J Am Soc Nephrol (2014) 25(3):443–53. doi: 10.1681/ASN.2013090950 PMC3935594 24408874

[47] Tenten V Menzel S Kunter U Sicking EM van Roeyen CR Sanden SK. Albumin is Recycled From the Primary Urine by Tubular Transcytosis. J Am Soc Nephrol (2013) 24(12):1966–80. doi: 10.1681/ASN.2013010018 PMC3839546 23970123

[48] Sarav M Wang Y Hack BK Chang A Jensen M Bao L. Renal FcRn Reclaims Albumin But Facilitates Elimination of IgG. J Am Soc Nephrol (2009) 20(9):1941–52. doi: 10.1681/ASN.2008090976 PMC2736763 19661163

[49] Greenblatt DJ Sellers EM Koch-Weser J. Importance of Protein Binding for the Interpretation of Serum or Plasma Drug Concentrations. J Clin Pharmacol (1982) 22(5-6):259–63. doi: 10.1002/j.1552-4604.1982.tb02671.x 7107972

[50] Oettl K Stauber RE. Physiological and Pathological Changes in the Redox State of Human Serum Albumin Critically Influence its Binding Properties. Br J Pharmacol (2007) 151(5):580–90. doi: 10.1038/sj.bjp.0707251 PMC2013999 17471184

[51] Maciazek-Jurczyk M Janas K Pozycka J Szkudlarek A Rogoz W Owczarzy A. Human Serum Albumin Aggregation/Fibrillation and its Abilities to Drugs Binding. Molecules (2020) 25(3):618. doi: 10.3390/molecules25030618 PMC7038104 32023900

[52] Sleep D Cameron J Evans LR. Albumin as a Versatile Platform for Drug Half-Life Extension. Biochim Biophys Acta (2013) 1830(12):5526–34. doi: 10.1016/j.bbagen.2013.04.023 23639804

[53] Hoogenboezem EN Duvall CL. Harnessing Albumin as a Carrier for Cancer Therapies. Adv Drug Deliv Rev (2018) 130:73–89. doi: 10.1016/j.addr.2018.07.011 30012492 PMC6200408

[54] Gurevich KG. Effect of Blood Protein Concentrations on Drug-Dosing Regimes: Practical Guidance. Theor Biol Med Model (2013) 10:20. doi: 10.1186/1742-4682-10-20 23506635 PMC3606132

[55] Watanabe H Imafuku T Otagiri M Maruyama T. Clinical Implications Associated With the Posttranslational Modification-Induced Functional Impairment of Albumin in Oxidative Stress-Related Diseases. J Pharm Sci (2017) 106(9):2195–203. doi: 10.1016/j.xphs.2017.03.002 28302542

[56] Anguizola J Matsuda R Barnaby OS Hoy KS Wa C DeBolt E. Review: Glycation of Human Serum Albumin. Clin Chim Acta (2013) 425:64–76. doi: 10.1016/j.cca.2013.07.013 23891854 PMC3795802

[57] Leblanc Y Berger M Seifert A Bihoreau N Chevreux G. Human Serum Albumin Presents Isoform Variants With Altered Neonatal Fc Receptor Interactions. Protein Sci (2019) 28(11):1982–92. doi: 10.1002/pro.3733 PMC6798133 31583777

[58] Martinez Fernandez A Regazzoni L Brioschi M Gianazza E Agostoni P Aldini G. Pro-Oxidant and Pro-Inflammatory Effects of Glycated Albumin on Cardiomyocytes. Free Radic Biol Med (2019) 144:245–55. doi: 10.1016/j.freeradbiomed.2019.06.023 31260731

[59] Brioschi M Gianazza E Mallia A Zoanni B Altomare A Martinez Fernandez A. S-Thiolation Targets Albumin in Heart Failure. Antioxid (Basel) (2020) 9(8):763. doi: 10.3390/antiox9080763 PMC7463808 32824562

[60] Abu-Qare AW Abou-Donia MB. Binding of Pyridostigmine Bromide, N,N-Diethyl-M-Toluamide and Permethrin, Alone and in Combinations, to Human Serum Albumin. Arch Toxicol (2002) 76(4):203–8. doi: 10.1007/s00204-002-0328-8 12029383

[61] Drugbank (2017). Available at: https://go.drugbank.com/drugs. (Accessed February 22, 2022).

[62] FDA. FY2015 Regulatory Science Research Report: Narrow Therapeutic Index Drugs (2017). Available at: https://www.fda.gov/industry/generic-drug-user-fee-amendments/fy2015-regulatory-science-research-report-narrow-therapeutic-index-drugs#:~:text=Narrow%20therapeutic%20index%20drugs%20are,or%20significant%20disability%20or%20incapacity. (Accessed February 22, 2022).

[63] Cacho Diaz B Flores-Gavilán P García-Ramos G. Myasthenia Gravis and its Comorbidities. J Neurol Neurophysiol (2015) 06(05):1000317. doi: 10.4172/2155-9562.1000317

[64] Robles NR Velasco J Mena C Polo J Angulo E Espinosa J. Increased Frequency of Microalbuminuria in Patients Receiving Statins. Clin Lipidol (2013) 8(2):257–62. doi: 10.2217/clp.13.5

[65] Basi S Fesler P Mimran A Lewis JB. Microalbuminuria in Type 2 Diabetes and Hypertension: A Marker, Treatment Target, or Innocent Bystander? Diabetes Care (2008) 31(Suppl 2):S194–201. doi: 10.2337/dc08-s249 18227485

[66] Oh SJ Dhall R Young A Morgan MB Lu L Claussen GC. Statins may Aggravate Myasthenia Gravis. Muscle Nerve (2008) 38(3):1101–7. doi: 10.1002/mus.21074 PMC2670554 18720508

[67] Miller M. Dyslipidemia and Cardiovascular Risk: The Importance of Early Prevention. QJM (2009) 102(9):657–67. doi: 10.1093/qjmed/hcp065 PMC2729130 19498039

[68] Charlier B Coglianese A De Rosa F de Grazia U Operto FF Coppola G. The Effect of Plasma Protein Binding on the Therapeutic Monitoring of Antiseizure Medications. Pharmaceutics (2021) 13(8):1208. doi: 10.3390/pharmaceutics13081208 34452168 PMC8401952

[69] Wojakowski E Cheruvil C Hassan A Holsen MR Chen L Rossi M. Albumin and Bleed Risk in Rivaroxaban Treated Patients. J Thromb Thrombolysis (2020) 50(4):1004–11. doi: 10.1007/s11239-020-02092-w 32279215

[70] Tincani E Mazzali F Morini L. Hypoalbuminemia as a Risk Factor for Over-Anticoagulation. Am J Med (2002) 112:247–8. doi: 10.1016/s0002-9343(01)00957-3 11893361

[71] Akirov A Masri-Iraqi H Atamna A Shimon I. Low Albumin Levels are Associated With Mortality Risk in Hospitalized Patients. Am J Med (2017) 130(12):1465 e11– e19. doi: 10.1016/j.amjmed.2017.07.020 28803138

[72] Weng YY Yang DH Qian MZ Wei MM Yin F Li J. Low Serum Albumin Concentrations are Associated With Disease Severity in Patients With Myasthenia Gravis. Med (Baltimore) (2016) 95(39):e5000. doi: 10.1097/MD.0000000000005000 PMC5265951 27684858

[73] Arques S. Serum Albumin and Cardiovascular Disease: State-Of-the-Art Review. Ann Cardiol Angeiol (Paris) (2020) 69(4):192–200. doi: 10.1016/j.ancard.2020.07.012 32797938

[74] Mace C Chugh SS. Nephrotic Syndrome: Components, Connections, and Angiopoietin-Like 4-Related Therapeutics. J Am Soc Nephrol (2014) 25(11):2393–8. doi: 10.1681/ASN.2014030267 PMC4214538 24854282

[75] Gerstein HC Mann JF Yi Q Zinman B Dinneen SF Hoogwerf B. Albuminuria and Risk of Cardiovascular Events, Death, and Heart Failure in Diabetic and Nondiabetic Individuals. JAMA (2001) 286(4):421–6. doi: 10.1001/jama.286.4.421 11466120

[76] Stehouwer CD Smulders YM. Microalbuminuria and Risk for Cardiovascular Disease: Analysis of Potential Mechanisms. J Am Soc Nephrol (2006) 17(8):2106–11. doi: 10.1681/ASN.2005121288 16825333

[77] Murai S Tanaka S Dohi Y Kimura G Ohte N. The Prevalence, Characteristics, and Clinical Significance of Abnormal Albuminuria in Patients With Hypertension. Sci Rep (2014) 4:3884. doi: 10.1038/srep03884 24457614 PMC3900920

[78] Roopenian DC Low BE Christianson GJ Proetzel G Sproule TJ Wiles MV. Albumin-Deficient Mouse Models for Studying Metabolism of Human Albumin and Pharmacokinetics of Albumin-Based Drugs. MAbs (2015) 7(2):344–51. doi: 10.1080/19420862.2015.1008345 PMC4623309 25654695

[79] Demant T Mathes C Gutlich K Bedynek A Steinhauer HB Bosch T. A Simultaneous Study of the Metabolism of Apolipoprotein B and Albumin in Nephrotic Patients. Kidney Int (1998) 54(6):2064–80. doi: 10.1046/j.1523-1755.1998.00204.x 9853272

[80] Koot BG Houwen R Pot DJ Nauta J. Congenital Analbuminaemia: Biochemical and Clinical Implications. A Case Report and Literature Review. Eur J Pediatr (2004) 163(11):664–70. doi: 10.1007/s00431-004-1492-z 15300429

[81] Sankaranarayanan S de la Llera-Moya M Drazul-Schrader D Phillips MC Kellner-Weibel G Rothblat GH. Serum Albumin Acts as a Shuttle to Enhance Cholesterol Efflux From Cells. J Lipid Res (2013) 54(3):671–6. doi: 10.1194/jlr.M031336 PMC3617942 23288948

[82] Bernardi M Angeli P Claria J Moreau R Gines P Jalan R. Albumin in Decompensated Cirrhosis: New Concepts and Perspectives. Gut (2020) 69(6):1127–38. doi: 10.1136/gutjnl-2019-318843 PMC7282556 32102926

[83] Hillege HL Fidler V Diercks GF van Gilst WH de Zeeuw D van Veldhuisen DJ. Urinary Albumin Excretion Predicts Cardiovascular and Noncardiovascular Mortality in General Population. Circulation (2002) 106(14):1777–82. doi: 10.1161/01.cir.0000031732.78052.81 12356629

[84] Li WC Mo LJ Shi X Lin ZY Li YY Yang Z. Antioxidant Status of Serum Bilirubin, Uric Acid and Albumin in Pemphigus Vulgaris. Clin Exp Dermatol (2018) 43(2):158–63. doi: 10.1111/ced.13289 29067729

[85] Fokkink WR Walgaard C Kuitwaard K Tio-Gillen AP van Doorn PA Jacobs BC. Association of Albumin Levels With Outcome in Intravenous Immunoglobulin-Treated Guillain-Barre Syndrome. JAMA Neurol (2017) 74(2):189–96. doi: 10.1001/jamaneurol.2016.4480 28027337

[86] Yang D Su Z Wu S Bi Y Li X Li J. Low Antioxidant Status of Serum Bilirubin, Uric Acid, Albumin and Creatinine in Patients With Myasthenia Gravis. Int J Neurosci (2016) 126(12):1120–6. doi: 10.3109/00207454.2015.1134526 26707693

[87] Yoshimoto Y Ishida S Hosokawa T Arawaka S. Assessment of Clinical Factors Affecting Outcome of Myasthenia Gravis. Muscle Nerve (2021) 64(1):90–4. doi: 10.1002/mus.27247 33885175

[88] Keller CW Pawlitzki M Wiendl H Lunemann JD. Fc-Receptor Targeted Therapies for the Treatment of Myasthenia Gravis. Int J Mol Sci (2021) 22(11):5755. doi: 10.3390/ijms22115755 34071155 PMC8198115

[89] Wolfe GI Ward ES de Haard H Ulrichts P Mozaffar T Pasnoor M. IgG Regulation Through FcRn Blocking: A Novel Mechanism for the Treatment of Myasthenia Gravis. J Neurol Sci (2021) 430:118074. doi: 10.1016/j.jns.2021.118074 34563918

[90] Smith B Kiessling A Lledo-Garcia R Dixon KL Christodoulou L Catley MC. Generation and Characterization of a High Affinity Anti-Human FcRn Antibody, Rozanolixizumab, and the Effects of Different Molecular Formats on the Reduction of Plasma IgG Concentration. MAbs (2018) 10(7):1111–30. doi: 10.1080/19420862.2018.1505464 PMC6291300 30130439

[91] Kiessling P Lledo-Garcia R Watanabe S Langdon G Tran D Bari M. The FcRn Inhibitor Rozanolixizumab Reduces Human Serum IgG Concentration: A Randomized Phase 1 Study. Sci Transl Med (2017) 9(414):eaan1208. doi: 10.1126/scitranslmed.aan1208 29093180

[92] Ling L Roy S Daly T Cochran E Tyler S Markowitz L. M281: A Therapeutic FcRn Blocking Antibody for Rapid Clearance of IgG and IgG Autoantibodies in Immune Cytopenias and Other Autoimmune Diseases. Blood (2015) 126(23):3472. doi: 10.1182/blood.V126.23.3472.3472

[93] Ling LE Hillson JL Tiessen RG Bosje T van Iersel MP Nix DJ. M281, an Anti-FcRn Antibody: Pharmacodynamics, Pharmacokinetics, and Safety Across the Full Range of IgG Reduction in a First-in-Human Study. Clin Pharmacol Ther (2019) 105(4):1031–9. doi: 10.1002/cpt.1276 PMC6587432 30402880

[94] Blumberg L Humphries JE Jones SD Pearce LB Holgate R Hearn A. Blocking FcRn in Humans Reduces Circulating IgG Levels and Inhibits IgG Immune Complex-Mediated Immune Responses. Sci Adv (2019) 12(5):eaax9586. doi: 10.1126/sciadv.aax9586 PMC6920022 31897428

[95] Yap DYH Hai J Lee PCH Zhou X Lee M Zhang Y. Safety, Tolerability, Pharmacokinetics, and Pharmacodynamics of HBM9161, a Novel FcRn Inhibitor, in a Phase I Study for Healthy Chinese Volunteers. Clin Transl Sci (2021) 14(5):1769–79. doi: 10.1111/cts.13019 PMC8504844 33742786

[96] Collins JC Jones L Snyder MM Sicard E Griffin P Webster L. RVT-1401, a Novel Anti-FcRn Monoclonal Antibody, is Well Tolerated in Healthy Subjects and Reduces Plasma IgG Following Subcutaneous or Intravenous Administration P5.2–079. Neurology (2019) 92.

[97] Ulrichts P Guglietta A Dreier T van Bragt T Hanssens V Hofman E. Neonatal Fc Receptor Antagonist Efgartigimod Safely and Sustainably Reduces IgGs in Humans. J Clin Invest (2018) 128(10):4372–86. doi: 10.1172/JCI97911 PMC6159959 30040076

[98] Robak T Kazmierczak M Jarque I Musteata V Trelinski J Cooper N. Phase 2 Multiple-Dose Study of an FcRn Inhibitor, Rozanolixizumab, in Patients With Primary Immune Thrombocytopenia. Blood Adv (2020) 4(17):4136–46. doi: 10.1182/bloodadvances.2020002003 PMC7479959 32886753

[99] Bril V Benatar M Andersen H Vissing J Brock M Greve B. Efficacy and Safety of Rozanolixizumab in Moderate to Severe Generalized Myasthenia Gravis: A Phase 2 Randomized Control Trial. Neurology (2021) 96(6):e853–e65. doi: 10.1212/WNL.0000000000011108 PMC8105899 33219142

[100] Werth VP Culton DA Concha JSS Graydon JS Blumberg LJ Okawa J. Safety, Tolerability, and Activity of ALXN1830 Targeting the Neonatal Fc Receptor in Chronic Pemphigus. J Invest Dermatol (2021) 141(12):2858–65 e4. doi: 10.1016/j.jid.2021.04.031 34126109

[101] Men CJ Kossler AL Wester ST. Updates on the Understanding and Management of Thyroid Eye Disease. Ther Adv Ophthalmol (2021) 13:25158414211027760. doi: 10.1177/25158414211027760 34263138 PMC8252358

[102] Newland AC Sanchez-Gonzalez B Rejto L Egyed M Romanyuk N Godar M. Phase 2 Study of Efgartigimod, a Novel FcRn Antagonist, in Adult Patients With Primary Immune Thrombocytopenia. Am J Hematol (2020) 95(2):178–87. doi: 10.1002/ajh.25680 PMC7004056 31821591

[103] Goebeler M Bata-Csörgõ Z De Simone C Didona B Remenyik E Reznichenko N. Treatment of Pemphigus Vulgaris and Foliaceus With Efgartigimod, a Neonatal Fc Receptor Inhibitor: A Phase II Multicentre, Open-Label Feasibility Trial. Br J Dermatol (2022) 186(3):429–39. doi: 10.1111/bjd.20782 34608631

[104] Howard JF Jr. Bril V Vu T Karam C Peric S Margania T. Safety, Efficacy, and Tolerability of Efgartigimod in Patients With Generalised Myasthenia Gravis (ADAPT): A Multicentre, Randomised, Placebo-Controlled, Phase 3 Trial. Lancet Neurol (2021) 20(7):526–36. doi: 10.1016/S1474-4422(21)00159-9 34146511

[105] Vyvgart. Prescribing Information (2021). Argenx (Accessed February 24, 2022).

[106] Vaccaro C Zhou J Ober RJ Ward ES. Engineering the Fc Region of Immunoglobulin G to Modulate *In Vivo* Antibody Levels. Nat Biotechnol (2005) 23(10):1283–8. doi: 10.1038/nbt1143 16186811

[107] Weflen AW Baier N Tang QJ Van den Hof M Blumberg RS Lencer WI. Multivalent Immune Complexes Divert FcRn to Lysosomes by Exclusion From Recycling Sorting Tubules. Mol Biol Cell (2013) 24(15):2398–405. doi: 10.1091/mbc.E13-04-0174 PMC3727932 23741050

[108] Nguyen-Cao TM Gelinas D Griffin R Mondou E. Myasthenia Gravis: Historical Achievements and the "Golden Age" of Clinical Trials. J Neurol Sci (2019) 406:116428. doi: 10.1016/j.jns.2019.116428 31574325

